# News and Coming Events

**Published:** 1908-08-22

**Authors:** 


### News and Coming Events.

The Prince of Wales has given £25 towards the proposed enlargement of the Launceston (Cornwall) Infirmary.

Mr. O. L. Addison, M.B., Lond., M.R.C.S., has been appointed surgeon to out-patients at the Hospital for Sick Children, Great Ormond Street.

In aid of the Hospital for Women, Soho Square, a Concert, promoted by Miss Annette Ellis, will be given at the Æolian Hall on October 31.

The German Society of Scientists and Medical Practitioners will hold its eightieth annual meeting this year at Cologne from September 20 to 26. Among the general addresses to be delivered is one by Professor Doflein, of Munich, on trypanosomes.

The eighth German medical excursion, consisting of 350 German physicians, conducted by Professor von Strümpell, the President; Dr. Oliven, First Secretary; and Dr. Bassenge, Secretary, will visit Ryde, in the Isle of Wight, on September 3. Visits will be paid to Ventnor and Cowes and to Osborne House.

Mr. Charles Marshall Kempe, M.R.C.S. Eng., L.S.A., who recently retired from the office of medical officer of health of Shoreham (Sussex), a position which he had held continuously for thirty-five years, was on August 4 presented by the Urban District Council with an illuminated address in recognition of his services.

The "Anti-Suicide Bureau" of the Salvation Army has recently completed eighteen months of existence. In the first year of the life of the bureau 1,125 applications were received, and since then it is stated that people have been seeking aid at the rate of about twenty a week. The bureau has several ex-soldiers on the books for whom it is trying to get work through the military employment organisation; while the clients of the bureau during the eighteen months have comprised clergymen, missionaries, military officers, doctors, solicitors, and representatives of almost every profession and trade.

Steps are being taken to commemorate the great services rendered to Manchester and district by the late Dr. Henry Ashby, the distinguished pediatrist. A committee has been formed to raise a fund with the object of carrying out this purpose. It is proposed that a research scholarship shall be founded for the promotion of the study and treatment of the diseases of children. It is felt that this object will commend itself as being an especially appropriate memorial of the life-work of Dr. Ashby, whose labours for the welfare of children are so widely known throughout the world. Subscriptions to the fund may be forwarded direct to Lloyd's Bank, King Street, Manchester ("Dr. Ashby Memorial Fund"), or to Mr. J. B. Close Brooks, honorary treasurer, Lloyd's Bank, King Street, Manchester; or Mr. J. Howson Ray, F.R.C.S., honorary secretary, 11 St. John Street, Manchester.

Professor Norman Dalton, M.D., Alfred Boyce Barrow, Esq., M.B., and Professor Albert Carless, M.B., M.S., have been elected Fellows of King's College, London.

The late Dr. H. J. Hunter, who died recently at Bath, has bequeathed the residue of his estate (estimated at between £15,000 and £20,000) to the funds of the University of Sheffield.

In connection with the 3rd Scottish General Hospital (Territorial) Dr. Donald James Mackintosh, M.V.O., transport officer Scottish Command, Glasgow Companies, Royal Army Medical Corps (Volunteers), has been appointed Lieutenant-Colonel.

The measures devised by the Governor of Uganda, Sir Henry Hesketh Bell, for combating the spread of sleeping-sickness are, according to Reuter's Agency, meeting with a considerable measure of success. During 1907 there were no new cases among Europeans, and the deaths among natives during the twelve months numbered less than 4,000. The whole of the population has been removed from the shores of the Victoria Nyanza, and it is hoped that the disease-carrying fly in that belt, if not reinfected, will gradually cease to be a source of danger. Several thousands of the sufferers from sleeping-sickness are being maintained in segregation camps, but the treatment by atoxyl is not proving of much avail. Consistent and vigorous action will be necessary for some years to come if sleeping-sickness is to be stamped out of the country.

At a meeting of representatives of temperance organisations interested in the International Anti-Alcohol Congress, which has been held during the past twenty years in various Continental towns, it was agreed to invite the Congress to meet next year in London for the first time. That invitation has been accepted, and arrangements are now being made for the twelfth Congress to be held during the week beginning July 18, 1909. The Congress, which is to last a week, will probably meet at the Imperial Institute, and the delegates will be present from nearly every country in the world. The Duke of Connaught, President of the Royal Army Temperance Association, will act as Honorary President, while the Dean of Hereford will be Chairman and the Hon. Mrs. Eliot Yorke Vice-Chairman of the Congress committee, a meeting of which is to be held on Friday, September 4, to receive reports from the various sectional committees. More than fifty temperance organisations are represented on the general committee, and the Vice-Presidents include the Archbishop of Canterbury, the Lord Chief Justice, Lord Carlisle, Lord Kinnaird, Mr. John Burns, M.P., Sir Victor Horsley, and Professor Sims Woodhead. The official address of the Congress committee is Paternoster House, Paternoster Row, E.C.

The St. Thomas's Hospital Old Students' dinner will take place at the Savoy Hotel, Strand, on Friday, October 2, at 7 o'clock for 7.30. The chair will be taken by T. Wakley, Esq. Messrs. J. E. Adams and M. A. Cassidy are the hon. secretaries. The price of the dinner, inclusive of stewards' fee, is 12s. 6d., to be paid at the Savoy Hotel.

---

The Incorporated Society for the Destruction of Vermin, whose headquarters are at 95 Wigmore Street, has decided to endeavour to stimulate rat destruction by offering an award for the best method of utilising rat skins; branches of the Society have been formed in India and Australia. The Society has decided to hold an exhibition next May and to issue a quarterly journal. It is now seeking to increase its guarantee fund in order to carry out its programme.

---

According to a return issued in the *London Gazette* of August 18, the number of outbreaks of anthrax reported in the United Kingdom in the week ended August 15 was seventeen, and the number of animals attacked was twenty-five. These figures compare unfavourably with those of the corresponding weeks of 1907, 1906, and 1905. In 1905 the cases numbered eight, and fourteen animals were attacked. The total number of cases in the thirty-three weeks of 1908 is returned at 727, and the number of animals attacked is 977, as against 719 and 950 in 1907. In 1906 there were 594 outbreaks, and 876 animals were attacked. The number of cases in 1905 was 633, and 877 animals were attacked.

---

We regret to announce the death of Mr. Harold Leslie Barnard, F.R.C.S., surgeon-in-charge of outpatients at the London Hospital, at the early age of forty-one. Mr. Barnard's death deprives the profession of one of her ablest members, who seemed destined to attain a most distinguished position in the world of surgery. Almost his whole professional life was spent in close connection with the London Hospital, and his loss will be felt deeply by students and staff alike. Mr. Barnard qualified in 1892 and held nearly every available house appointment. He took first-class honours in the M.B., B.S. Lond. examination in 1895, and obtained the F.R.C.S. Eng. in the same year, while in 1896 he received the M.S. degree. For several years he was Demonstrator of Physiology in the Medical School of the London Hospital, and he also served as Assistant Demonstrator of Anatomy and Surgical Tutor. As a physiologist his work is well known. At the time of his death Mr. Barnard was out-patient surgeon to the London Hospital and Demonstrator of Surgical Pathology, and he had previously held the post of assistant-surgeon to the Metropolitan Hospital. His principal contributions to medical literature were upon topics related to abdominal surgery, and the article "Intestinal Obstruction" in the last edition of Allbutt's "System of Medicine" was from his pen.

At the opening of the winter session at King's College, London, Professor Myers, M.A., M.D., will deliver an introductory lecture on "The Aims and Position of Experimental Psychology" on Wednesday, September 30, at 4 p.m. On Thursday, October 1, the prizes in the Medical Faculty will be distributed by Professor A. Macalister, LL.D., F.R.S., who will give an introductory address on "Fifty Years of Medical Education," at 4 p.m.

---

Dr. Lucas-Championniere, the celebrated French surgeon, attended at the Royal College of Surgeons of England, on August 18 and signed the Roll of Honorary Fellows in pursuance of his election by the Council. This order of Fellows was instituted in the year 1900, to celebrate the centenary of the Fellowship of the College, and includes the names of the King, the late Marquis of Salisbury, the Earl of Rosebery, Earl Roberts, and distinguished foreign surgeons. Dr. Lucas-Championnière has held the post of senior surgeon at the Beaujon Hospital; and, besides being a member of the Paris Academy of Medicine, is also consulting surgeon to the Hôtel Dieu, Paris.

---

Preparations are being made by the New York Committee of the International Congress on Tuberculosis to hold a clean-milk exhibit during the three weeks of the Tuberculosis Exhibition at Washington, from September 21 to October 12, 1908. The co-operation of a number of dairy owners has been obtained, and we learn from the *Medical Record* that the exhibit will include photographs of dairies, statistical charts, Petri plates of the bacteriology of milk, and illustrations of tuberculin tests in cattle, and will also have a small working dairy with tuberculin tested cow, skilled attendant, sanitary utensils, shipping cases, and all sanitary appliances for the marketing of a clean milk. In view of the fact that Mr. Nathan Straus is preparing for the exhibition a duplicate of the pasteurisation plants already erected by him in Heidelberg, Brussels, and Berlin, the exhibition at Washington will afford an opportunity for a comparative study of the two methods of marketing milk, namely (1) pasteurised, and (2) unpasteurised but clean.

---

### THE GRADUATES' MEDICAL SCHOOLS.

DIARY FOR WEEK AUGUST 24 TO 29.

### THE POST-GRADUATE COLLEGE, West London Hospital, Hammersmith, W.

**At 12 noon.**

**August 24,** Dr. Low, **Pathological Demonstration.**

**At 5 p.m.**

**August 25,** Dr. Abraham, **Cases of Skin Diseases.**

**At 5 p.m.**

**August 26,** Mr. Pardoe, **The Diagnosis of Surgical Diseases of the Kidney.**

---

THE HOSPITAL August 22, 1908.

